# A revision of *Syngalepsus* Beier, with the description of two new species from the Central African Republic and Malawi (Mantodea, Tarachodidae)

**DOI:** 10.3897/zookeys.802.26622

**Published:** 2018-12-04

**Authors:** Nicolas Moulin

**Affiliations:** 1 82, route de l’école, Saveaumare, 76680, Montérolier, France Unaffiliated Montérolier France

**Keywords:** Africa, Dictyoptera, Dzanga-Ndoki National Park, Key, Malawi, Tarachodinae

## Abstract

The praying mantis subgenus Syngalepsus Beier, 1954 occurs in sub-Saharan region and represents the least diverse subgenus of *Galepsus* in Africa (Ehrmann 2002). All species included within the subgenus Syngalepsus are comprehensively revised with a distribution stretching from North of Congo Basin to South Africa. Two new species of Galepsus (Syngalepsus) Beier, 1954 (Mantodea, Tarachodidae) from the Central African Republic (CAR) and Malawi are described. Among several *Galepsus* specimens collected during scientific expeditions of SANGHA, Biodiversité en Terre Pygmée, in CAR’s Dzanga-Ndoki National Park, a specimen differed by genitalia conformation and other morphological characters. Two specimens collected in Malawi proved to differ by genitalia morphology. Galepsus (Syngalepsus) bucheti**sp. n.** and Galepsus (Syngalepsus) dudleyi**sp. n.** are described. An identification key for the six species of the subgenus is provided.

## Abstract (fr)

Les mantes du sous-genre *Syngalepsus* Beier, 1954 se rencontrent en région sub-saharienne et représentent le sous-genre le moins diversifié des *Galepsus* en Afrique (Ehrmann 2002). L’ensemble des espèces du sous-genre *Syngalepsus* sont révisées avec leur distribution s’étirant du nord du bassin du Congo à l’Afrique du Sud. Deux nouvelles espèces de Galepsus (Syngalepsus) Beier, 1954, provenant de République centrafricaine et du Malawi (Mantodea, Tarachodidae) sont décrites. Parmi les différents spécimens de *Galepsus* rapportés des missions scientifiques du programme SANGHA, Biodiversité en Terre Pygmée, situées au sein du Parc National Dzanga-Ndoki, un individu diffère des autres par la conformation de sa morphologie et ses genitalia. Durant des recherches au Muséum national d’Histoire naturelle, Paris, deux spécimens collectés au Malawi diffèrent par la morphologie de leurs genitalia. Galepsus (Syngalepsus) bucheti**n. sp.** et Galepsus (Syngalepsus) dudleyi**n. sp.** sont décrites. Une clé dichotomique est proposée pour l’identification des six espèces du sous-genre *Syngalepsus* maintenant connues.

## Introduction

SubgenusSyngalepsus was erected by Max Beier in 1954 to organise some species from the genus *Galepsus* Stål, 1876: G. (S.) bipunctatus described in 1931 and G. (S.) denigratusBeier 1954. Beier also described G. (S.) birkenmeierae in 1969. Kaltenbach described a fourth species, G. (S.) beieri, from South Africa in 1996. In total, four species are currently known (Ehrmann 2002, Otte and Spearman 2005). All species of the subgenus have the vertex, straight or slightly arched, without bump or incision near the eyes and a right phallomere with the posterior apical region rounded and toothless, unlike *Onychogalepsus*. The prosternum has two circular black spots, more or less covered by a black patch. Three of the species are present in Southern Africa: South Africa (Natal) for G. (S.) beieriKaltenbach (1996: 233); Mozambique and South Africa for G. (S.) bipunctatusBeier (1931: 3) (Type species) and Malawi for G. (S.) birkenmeieraeBeier (1969b: 201). The fourth species, G. (S.) denigratusBeier (1954: 19), was collected in Angola (Beier 1969a), Gabon, Republic of Congo, Democratic Republic of Congo and Uganda (Beier 1957, Roy 1968).

The discovery of a specimen in CAR that seems close to *Syngalepsus* led me to gather all the existing information concerning this subgenus (Moulin et al. 2017). The old descriptions have been revised and the figures of the genitalia grouped. Examination of its morphology and that of other specimens preserved in the collection has highlighted a second new species, native to Malawi. This paper aims to provide additional knowledge about Mantodea from Africa. Here we provide details about morphology and distribution of known species of the subgenus Syngalepsus and describe two new species.

### Taxonomy

After the examination of several specimens of *Galepsus* genus in the Muséum national d’Histoire naturelle, Paris (MNHN) and a bibliographic survey (Beier 1931, 1954, 1957, 1969a, 1969b; Roy 1968; Kaltenbach 1996, 1998; Ehrmann 2002; Otte and Spearman 2005), the genus Galepsus (Syngalepsus) appears to include six species. Two of these are unknown and are described in this subgenus revision. The genitalia were illustrated from types for all existing species, which served as the central distinguishing feature for the treated species.

## Materials and methods

During the scientific expedition named “SANGHA, Biodiversité en terre Pygmée” (2012) in CAR, some *Galepsus* individuals were collected. Two species were identified: G. (Galepsus) globiceps Beier, 1942 and G. (Galepsus) laticeps Werner, 1907. But one specimen was atypical of the others and its genital characters were similar to those of *Syngalepsus*. In my comparisons to collection specimens, two undetermined specimens from Malawy were found in the material of the Muséum national d’Histoire naturelle, Paris, France. The specimens exhibit the diagnostic characters of the subgenus Syngalepsus (sensu Beier 1954) and differ from the RCA specimen and the four known species.

The specimens were photographed with a Dynax 5D Konica Minolta camera and a Leica MC 120 HD camera mounted on a S8APO Leica stereomicroscope. The freeware Combine ZP (Hadley 2008) was used to process images.

Taxonomy relied on Mantodea Species File website (Otte et al. 2018) as well as recent works on praying mantis molecular phylogenetics (Svenson and Whiting 2009, Wieland 2013, Roy 2014, Svenson et al. 2015). All morphological descriptions and measurements (in millimeters) refer to the material studied here unless explicitly stated otherwise. Terminology follows Brannoch et al. (2017) and Shcherbakov (2017). A total of 12 measurement classes were captured including:

1 Body length = length of body from central ocelli to posterior tip of wing or abdomen (intraspecifically variable measurement, primarily for general size estimation).

2 Forewing length = from proximal margin of axillary sclerites to distal tip of the discoidal region.

3 Pronotum length = from anterior margin to posterior margin.

4 Pronotum width = from lateral margins at the widest point, the supra-coxal bulge.

5 Head width = from lateral margins of the eyes at widest point.

6 Prothoracic coxae length = from proximal margin abutting pronotum to trochanter.

7 Prothoracic femur length = from proximal margin abutting trochanter to distal margin of genicular lobe.

8 Prothoracic tibiae length = from distal margin of genicular lobe to distal terminal spur.

9 Prothoracic femur width = at the widest point.

10 Anteroventral femoral spine count = all inner marginal ridge spines but excluding the genicular spine.

11 Anteroventral tibial spine count = all inner marginal ridge spines but excluding the distal terminal spur.

12 Posteroventral tibial spine count = all outer marginal ridge spines but excluding the distal terminal spur.

List of abbreviations:

**age** anterior groove;

**apa** posterior process of phalloid apophysis;

**bm** medial arm of sclerite R1A;

**fda** main lobe of the right phallomere;

**L2** sclerite L2;

**L4A** sclerite L4A;

**L4B** sclerite L4B;

**ldp** left distal process;

**paa** apical process;

**pda** distal process;

**pia** sclerotization pia;

**pva** sclerotization pva;

**R1A** sclerite R1A;

**R3** sclerite R3.

A map was created in QGIS 3.0.0. with administrative areas from GADM, the database of Global Administrative Areas website (https://gadm.org/).

**Figure 1.**
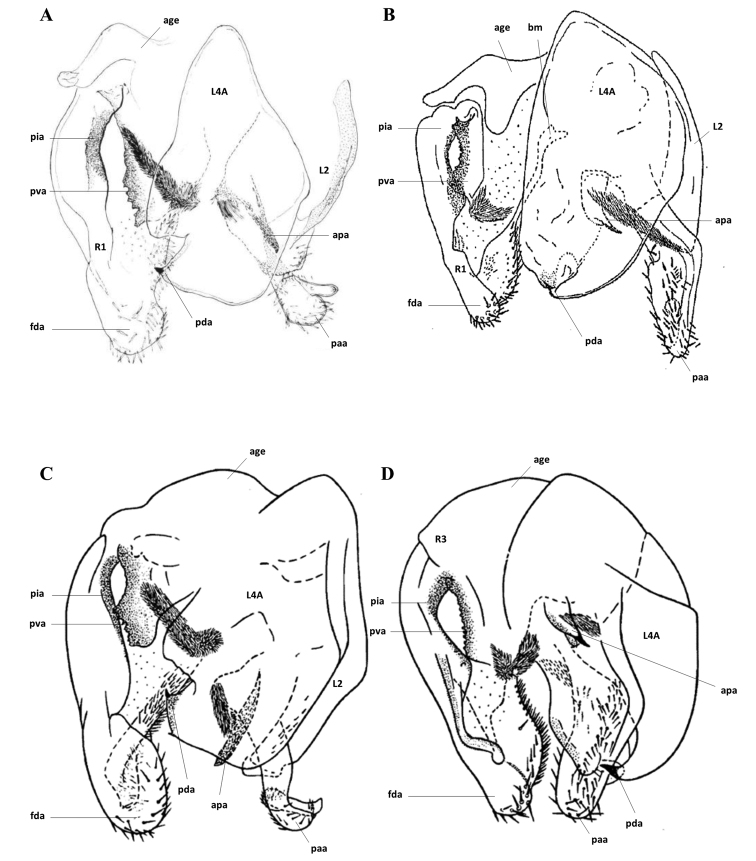
Genitalia: **A**Galepsus (S.) bipunctatus (modified from Kaltenbach 1996) **B**Galepsus (S.) denigratus (modified from Beier 1957) **C**Galepsus (S.) bipunctatus (modified from Beier 1954) **D**Galepsus (S.) denigratus (modified from Beier 1954). No scale. For abbreviations see Brannoch et al. (2017) and the text.

**Figure 2.**
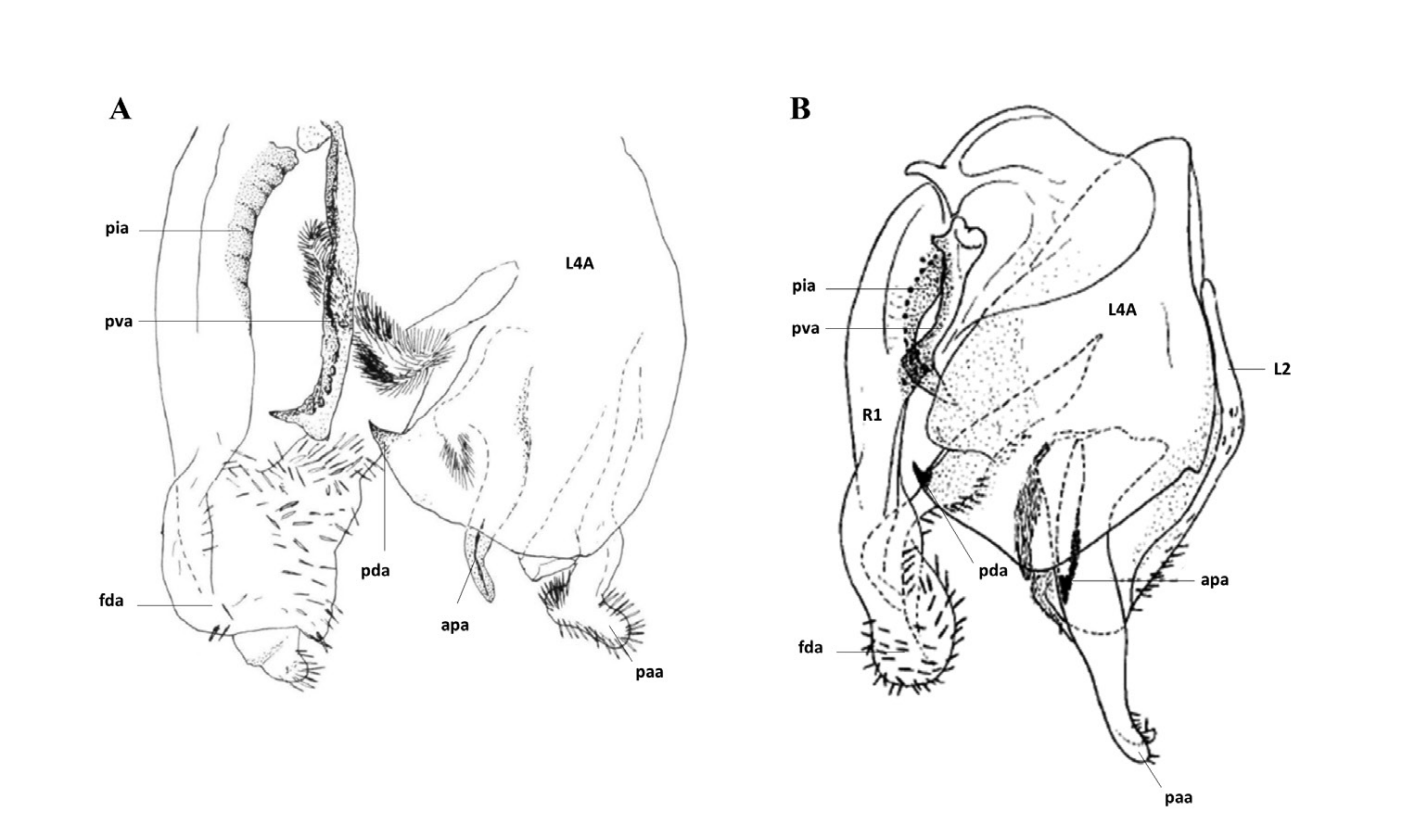
Genitalia: **A**Galepsus (S.) beieri (modified from Kaltenbach, 1996) **B**Galepsus (S.) birkenmeirae (modified from Beier 1969b). No scale. For abbreviations see Brannoch et al. (2017) and the text.

As part of the revision of *Syngalepsus*, the descriptions of the four known species are repeated here, according to the original texts, translated into English. The questioning of the identity of the female allotype referred to G. (S.) denigratus by Beier between 1957 and 1969 is re-evaluated here with the examination of two females, conserved at the MNHN, from the Republic of the Congo. The genitalia illustrated in the original documents are reproduced here (Figs 1, 2).

## Results

### Galepsus (Syngalepsus) bipunctatus

Taxon classificationAnimaliaMantodeaTarachodidae

Beier, 1931

Figures 1A , 6A , 7A , 8

Galepsus bipunctatus : Beier 1931: 3; Beier 1954: 19; Kaltenbach 1996: 233; Kaltenbach 1998: 40; Ehrmann 2002: 154; Otte and Spearman 2005: 336.

#### Holotype.

Male deposited in ZMUH Hamburg; Type locality: Quilimane, Mozambique. **Paratype**: Male deposited in NHM Wien.

#### Diagnosis.

Pronotum with a blackened median line. Right phallomere without a process at the apex of the main lobe (fda); apical process on left phallomere (paa) rounded at the apex, having a small lateral process unlike other species.

#### Original description of *Galepsusbipunctatus* by Beier (1931: 3).

“*Galepsusbipunctatus* n. sp. ♂. Gelblichbraun. Frontalschild etwas breiter als hoch. Scheitel leicht gerundet, ohne Höckerchen neben den Augen. Augen flach, seitlich kaum konvex, fast parallelseitig, mit deutlich ausgeprägtem, schmal abgerundetem oberen Eck. Der Scheitel ohne Winkel in den oberen Rand der Augen übergehend. Pronotum gut 2,5mal so lang als breit, fast parallelseitig, vorne kaum breiter als hinten, die Supracoxalerweiterung nicht ausgeprägt. Prosternum mit zwei runden, scharf, begrenzten schwarzen Punkten basal von der Mitte. Elytren kürzer als das Abdomen, hyalin. Alae hyalin, nur die Basis sehr leicht angeraucht und etwas irisierend. Vordercoxen einfarbig, basal leicht gebräunt. Trochanter ohne Fleck. Vorderfemora vollkommen einfarbig gelb, die Dornen nur an der Spitze dunkel. Tibien und Tarsenglieder einfarbig. Cerci flach, das letzte Glied lang und schmal, etwa viermal so lang als breit. Körperlänge 29 mm, Pronotum 6,8 mm, breit 2,5 mm, Metazone 4,3 mm, Elytren 18 mm.”

#### Translation.

“*Galepsusbipunctatus* n. sp. ♂. Yellowish brown. Lower frons slightly wider than high. Vertex slightly rounded, without juxtaocular bulges near the eyes. Eyes flat, hardly convex on the sides, almost parallel, with a pronounced, narrow rounded upper corner. The vertex without angle merging into the upper edge of the eyes. Pronotum more than 2.5 times as long as wide, margins almost parallel, scarcely wider at the front than at the back, the lateral pronotal expansion not pronounced. Prosternum with two rounded, sharp, limited black spots, basal from the middle. Forewings shorter than abdomen, not coloured. Hindwings not coloured, only the base slightly smoked and slightly iridescent. Forecoxae monochrome, slightly brown at the base. Trochanter without colour spot. Forefemora yellow, anteroventral femoral spines only dark at the apex. Tibiae and tarsi monochrome. Cerci flat, the distal cercomeri long and narrow, about 4 times as long as wide. Body length 29 mm, pronotum length 6.8 mm, width of pronotum 2.5 mm, metazone length 4.3 mm, forewing length 18 mm.”

### Galepsus (Syngalepsus) denigratus

Taxon classificationAnimaliaMantodeaTarachodidae

Beier, 1954

Figures 1B , 1D , 6B , 6D , 6E , 6F , 7B , 7D , 7E , 8

Galepsus (Syngalepsus) denigratus : Beier 1954: 19; Beier 1957: 141; Roy 1968: 318; Ehrmann 2002: 154; Otte and Spearman 2005: 336.

#### Holotype.

Male deposited in NHM Wien; Type locality: Lemfu, Democratic Republic of Congo. **Allotype**: Female deposited in Musée Royal de l’Afrique Centrale, Tervuren, Belgium. **Type locality**: Shamba, Kasai, Republic of the Congo.

#### Material examined.

2 females: Republic of the Congo, Voka, 4°40'16"S, 14°40'25"E, 611 m, end of year 1979 and 02.III.1980, Onore G. col. (MNHN).

#### Diagnosis.

One of the largest species of the subgenus Syngalepsus. Close to G. (S.) bipunctatus but larger. Prosternum largely blackened. Hind wings brown. Genitalia mainly different from other species by apical process (paa) of left phallomere more massive; with many large bristles.

#### Original description of Galepsus (Syngalepsus) denigratus by Beier (1954: 19).

“Galepsus (Syngalepsus) denigratus n. sp. Körper dorsal einfarbig gelblichbraun. Frontal schild ein wenig breiter als hoch, dorsal flach gerundet. Scheitel flach gewölbt, ganzrandig, nur in der Nähe der Augen mit einer seichten Furche. Augen länglich, flach, mit breit verrundetem Dorsaleck. Fühler einfarbig bräunlich. Pronotum schlank, etwa 3 mal so lang wie breit, die Metazone nur sehr wenig schmäler als die Prozone, Supracoxalerweiterung kaum angedeutet. Elytren des Männchens das Abdomenende nicht ganz erreichend, ziemlich stark rauchbraun getrübt, subhyalin, die Längs- und Queradern bräunlich, die falschen Längsadern weitgehend erhalten, aber zart. Alae wie die Elytren. Prosternum mit Ausnahme der Seitenränder und des Basalteiles in der Metazone geschwärzt, in dieser Schwärzung jedoch noch zwei tiefschwarze, kreisrunde Makeln erkennbar. Fangbeine medial einfarbig rötlich gelbbraun, der Trochanter lateral mit einer schwarzen Punktmakel. Vorderfemora mit 4 Aussendornen. Vordertibien mit 10 Aussen- und 11 Innendornen. Mittel- und Hinterbeine einfarbig, nicht punktiert, die Tarsenglieder apical schmal angedunkelt. Männliches Genitalorgan: Rechter Epiphallus stumpf, apikal abgerundet und medial bis zum Apex mit Spinderlborsten besetzt; linker Epiphallus ebenfalls stumpf und parallelseitig, der Apikalteil nicht abgebogen; linkes Basalsklerit klaunenförmig, mit kräftigen Endhaken; Hypophallus reduziert, weichhäutig; Phallus kurz. Körper L. ♂ 30 mm; Kopf B. 3 mm; Pronotum L. 7 mm, B. 2,2 mm; Elytren L. 19,5 mm.”

#### Translation.

“Galepsus (Syngalepsus) denigratus n. sp. Body dorsally monochrome yellowish brown. Lower frons slightly wider than high, dorsally flat rounded. Vertex flat arched, entire, only near the eyes with a shallow furrow. Eyes oblong, flat, with dorsal part broad and rounded. Antennae monochrome brownish. Pronotum slender, about 3 times as long as wide, metazone only very slightly narrower than prozone, supracoxal sulcus hardly indicated. Forewings of the male not quite reaching the abdomen apex, rather cloudy smoky-brown, clear, the longitudinal (anterior cubitus) and transverse veins brownish, the false longitudinal veins (media and radius) largely preserved, but tender. Hind wings similar to forewings. Prosternum blackened except lateral and basal boarder of metazone, but in this blackening there are two deep-black, circular spots. Fore legs monochrome with reddish ventral surface, trochanter with a lateral black spot. Fore femora with 4 posteroventral femoral spines. Fore tibia with 10 posteroventral tibial spines and 11 anteroventral tibial spines. Meso- and metathoracic legs monochrome, not punctuated, segments of the tarsi darkened apically. Male genitalia: Right phallomere blunt, main lobe (fda) apically rounded, with thick bristles near the middle. Apical process (paa) (titillator) blunt, with parallel sides, with its apex not bent; sclerite L4B claw-shaped, with strong end hook; ventral phallomere reduced, membranous. Posterior process of phalloid apophysis (apa) short. Body length ♂ 30 mm; width of head 3 mm; pronotum length 7 mm; width of pronotum 2.2 mm; hindwings length 19.5 mm.”

#### Additional description of Galepsus (Syngalepsus) denigratus by Beier (1957: 141).

“Zur Beschreibung des männlichen Kopulationsorgans ist folgendes nachzutragen: Der linke Epiphallus hat dorsal einen oralwärts gerichteten scheibenförmigen Anhang, medial einen stumpfen Haken; sein Basalsklerit ist kurz; der Phallus ist kurz, pfriemenförmig, mit relativ grossem Basalack; der Hypophallus hat einen derben, kahnförmigen Lateralteil und einen häutigen Lobus. Das Endglied der Cerci ist in der Regel lang und schmal. – Körper L. ♀ 26–30 mm; Kopf B. 2,9–3,1 mm; Pronotum L. 6,9–7 mm, B. 2,2 mm; Elytren L. 17,5–19,5 mm. Das Weibchen dieser Art, das in einem Stück (Allotype) von Kasai, Shamba, vorliegt, war noch unbekannt. Es ist dorsal fast einheitlich braun gefärbt, nur der Scheitelbogen ist geschwärzt und die Metazone des Pronotums trägt zwei grosse schwarze Flecke. Dorsalrand des etwas queren Frontalschildes fast gerade. Scheitel flach gewölbt. Elytren schwärzlich-rötlichbraun, ventral entlang dem R geschwärzt. Alae grösstenteils schwärzlich, mit rötlichbraunen Rändern. Prosternum wie beim Männchen. Vordercoxen präapikal mit schwärzlicher Querbinde. Trochanter medial grösstenteils schwarz. Femur medial in der Ventralhälfte Schwarz, mit schwarzen Dornen. Vordertibien medial bräunlich, die Tarsenglieder geschwärzt. Fang- und Schreitbeine lateral bezw, dorsal braun punktiert. Supraanalplatte breit dreieckig, gekielt. – Körper L. 27 mm; Kopf B. 3,3 mm; Pronotum L. 7 mm, B. 2,7 mm; Elytren L. 4 mm. – Es ist in der Färbung dem Weibchen von femoratus G.-Tos aus dem Betschuanaland sehr ähnlich, unterscheidet sich aber von diesem durch helle Vordertibien und die Körpermasse, vor allem durch schlankeres Pronotum und relative kürzere Elytren.”

#### Translation.

“To describe the male genitalia, the following is to be added: The left phallomere has a dorsally directed disc-shaped appendage (L4B), medially a blunt hook; sclerite L4A is short; the posterior process of phalloid apophysis (apa) is short, belt-shaped, with a relatively large basal coat; ventral phallomere has a rough, posterior lateral part and a membranous lobe. Distal cercomere are usually long and narrow. – Body length ♀ 26–30 mm; width of head 2,9–3,1 mm; pronotum length 6,9–7 mm; width of pronotum 2,2 mm; forewing length 17,5–19,5 mm.

The female of this species, of which there is one specimen (allotype) collected at Kasai, Shamba, was previously unknown. It is dorsally almost uniformly brown, only the vertex is blackened and metazone carries two large black spots. Dorsal margin transverse, lower frons almost straight. Vertex flat arched. Forewings blackish-reddish brown, blackened ventrally along anterior radius. Hindwings mostly blackish, with reddish brown edges. Prosternum as in the male. Forecoxae with blackish pre-apical bandage. Trochanter mostly black. Forefemora half black in ventral face, with black thorns. Fore tibiae half brownish, tarsus blackened. Meso- and metathoracic legs lateral and dorsal brown dotted. Supra-anal plate wide, triangular and keeled. – Body length 27 mm; width of head 3,3 mm; pronotum length 7 mm; width of pronotum 2,7 mm; forewings length 4 mm. It is very similar in colour to the female of Galepsus (Onychogalepsus) femoratus Giglio-Tos collected from Bechuana land (Botswana), but differs from it in terms of its bright fore tibiae and body mass, above all by a slimmer pronotum and relatively shorter forewings.”

The female symbol in the description of Beier, in 1957, is a mistake. He wanted to indicate the measurements for the males.

#### Additional description of Galepsus (Syngalepsus) denigratus by Beier (1969a: 21).

“Das von mir seinerzeit (1957) für das Weibchen von denigratus gehaltene Exemplar gehört offensichtlich nicht dieser Art an. Es liegen nun 4 Weibchen vor, die ich mit Bestimmtheit dieser Art zuzählen möchte. Sie lassen sich folgendermassen charakterisieren: Weibchen. Ober- und Unterseite einschliesslich der kurzen Elytren braun, der Scheitel in Längsstreifen schwach angedunkelt, Occiput bisweilen mit einem schwarzen Punkt; Pronotum mit zwei Paaren kleiner brauner Punkte in der vorderen Hälfte der Metazone, Prosternum wie beim Männchen, meist jedoch etwas breiter geschwärzt; Vordercoxen medial mit zwei schwarzen Apikalmakeln, Trochanter mit schwarzer Medialmakel, Vorderfemora medial vor und hinter der Krallenfurche mit einer grösseren schwarzen Makel, weiter distal mit einigen braunen Punktmakeln, an der Basis der hellen grossen Innendornen ebenfalls mit je einer braunen Punktmakel; Beine braun gesprenkelt. Seiten des Pronotums äusserst fein körnchenförmig gezähnelt. – Körper-L. 32–34 mm; Kopf-B. 3,8–4 mm; Pronotum-L. 7,5–8 mm, B. 2,9–3,1 mm; Elytren-L. 4,5–5 mm; Hinterfemora 6–6,5 mm. – Die Weibchen sind an der charakteristischen Fleckung der Voderbeine leicht kenntlich. Die Art ist neu für Angola. Sie war bisher nur aus dem Congo bekannt.”

#### Translation.

“The specimen I held at that time (1957) of the female *denigratus* obviously does not belong to this species. There are now 4 females that I want to count with certainty of this kind. They can be characterised as follows: Female. The upper and lower side including stigma of forewings, brown, the apex of the head dimly darkened in longitudinal stripes, gena sometimes with a black dot; pronotum with two pairs of small brown dots in the anterior half of the metazone, prosternum as in the male, but usually a little more blackened; fore coxae with two apical black spots, trochanter with a medial black spot, forefemora with a, medial and behind the claw furrow, larger blackish spot, further distal with a little brown spot, at the base of the larger anteroventral femoral spines also each with a brown spot; Legs speckled brown. Margins of pronotum extremely fine granulated serrated. – Body length 32–34 mm; width of head 3,8–4 mm; pronotum length 7,5–8 mm; width of pronotum 2,9–3,1 mm; forewings length 4,5–5 mm; metathoracic femora length 6–6,5 mm. – The females are easily recognizable by the characteristic patch on the forelegs.

The species is new to Angola. It was previously known only from the Congo.”

Note: The female described in 1957 from Shamba in Kasai (Republic of the Congo) is confirmed to be G. (S.) denigratus, after examination.

#### Redescription of female.

Fine *body*, brown, length 33.0 mm from head to apex of abdomen. Wings reduced, brown or more or less bicoloured according to examined specimens.

*Head*. Vertex slightly convex, brownish at the top. Parietal sulcus well marked. A brown-black spot on the vertex, against the parietal sulcus, near the juxtaocular bulge. Lower frons almost square, slightly wider than long. Eyes slightly prominent on the side, almost square head, front view. Antennae broken in both examined females. Maxillary palps with the base of the last segment brown-black.

*Thorax*. Pronotum with prozone almost as wide as metazone. Two small depressions, stained brown, on each side of the midline of the metazone, behind the supracoxal sulcus. Two parallel black spots in the middle of the metazone; closer to the edge than the median line. Two small black-brown bands, parallel to the median line, near the posterior border. Margin of pronotum serrated along its entire length. Prosternum with a large black-brown spot, not reaching the edges of the pronotum. Two depressions characteristic of the subgenus, not very visible because of this dark spot.

*Fore legs*. Femora with 12 anteroventral femoral spines, 4 posteroventral femoral spines, 4 discoidal spines; tibiae with 11–12 anteroventral tibial spines, 11–12 posteroventral tibial spines. Coxae with a big black-brown spot at the apex, in the posterior margin and a smaller one in a more anterior position. Trochanter with a smaller, black-brown spot, very close to the junction with the femora. Anteroventral face of femora with several dark brown spots of various sizes and shapes forming a band along the ventral half. Dark brown spots at the base of the big spines. Apex of anteroventral femoral spines dark brown. Tibiae with a darker longitudinal line inside. Meso- and metathoracic legs stained with dark-brown little spots as forelegs. *Wings*. Forewings and hindwings, short, not reaching the beginning of the 1^st^ abdominal segment. Forewings light brownish red, with a dark spot taking all the discoidal area. Costal area of the same colour as the first part of the discoidal area. Hind wings brownish. *Abdomen*. Stained with brown markings. Triangular supra-anal plate, as wide as the abdomen. Cerci relatively long, 4.5 mm, flattened, the last two distal cercomeri longer than wide.

*Measurements* (mm). Body length 30.2–33.0, pronotum length 7.2–8.2, fore wings length 4.1–5.0, fore coxae length 4.0–4.1, fore femora length 4.9–5.4, fore tibiae length 3.4–3.8; width of pronotum 2.6–2.7, width of head 3.4–3.9, width of fore femora 1.4–1.6.

### Galepsus (Syngalepsus) birkenmeierae

Taxon classificationAnimaliaMantodeaTarachodidae

Beier, 1969

Figures 2B , 6G , 7G , 8

Galepsus (Syngalepsus) birkenmeierae : Beier 1969a: 201; Ehrmann 2002: 154; Otte and Spearman 2005: 336.

#### Holotype.

Male deposited in NMW Vienne. **Type locality**: Fort Johnston, Malawi.

#### Diagnosis.

The smallest species of the subgenus Syngalepsus. Very close to G. (S.) bipunctatus by genitalia conformation, but smaller, head wider, eyes more rounded laterally, forewings shorter and prosternum with a black patch.

#### Original description of Galepsus (Syngalepsus) birkenmeierae by Beier (1969: 201).

“Galepsus (Syngalepsus) birkenmeierae n. sp. ♂. Relativ klein. Allgemeinfärbung gelblich braun, dunkler braun gesprenkelt. Kopf verhältnismäßig breit, viel breiter als das Pronotum. Scheitel fast gerade. Augen seitlich ziemlich stark gebaucht, dorsal breit abgerundet, ohne Kerbe in den Scheitel übergehend. Frontalschild deutlich etwas breiter als hoch. Fühler basal hell, distalwärts allmählich dunkler. Pronotum fast 3mal länger als breit, dunkelbraun gefleckt oder mit dunkler Medianlinie, die Metazone nur wenig schmäler als die Prozone. Prosternum mit großem, ovalem schwarzen Fleck, der bisweilen noch die für die Untergattung charakteristischen paarigen schwarzen Makeln erkennen läßt. Elytren das Abdomen nicht ganz bedeckend, ebenso wie die Alae leicht angeraucht, das Costalfeld mit hellen, das Discoidalfeld mit braunen Adern, die Queradern ziemlich derb, im Medialis- und Radius-Bereich an den falschen Längsadern wie diese hell und daher unterbrochen erscheinend. Fangbeine medial gelbbraun, nur das Femur mit kleiner schwarzer Basalmakel, lateral braun gesprenkelt, der Trochanter lateral mit drei schwarzen Makeln. Vordercoxen den Hinterrand des Prosternums fast erreichend. Mittel- und Hinterbeine dicht braun gesprenkelt. Cerci flach. Kopulationsorgan: Hypophallus mit kurzer medialer Endklaue, kleinem Subapikalzähnchen und breit verrundetem, in der Anlage rechtwinkeligem Lateraleck; rechter Epiphallus stumpf, am Ende leicht verdickt, mit kräftigen Spindelborsten; rechte Apophysenlippe mit einer Reihe granulierter Zäpfchen, linke Apophysenlippe mit stumpfem Basalzahn und gehöckertem Apikallobus; linker Epiphallus schlank, terminal stumpf gegabelt; Pseudophallus glatt, scharf zugespitzt, mit lang behaartem Basalsack. - Körper-L. 24–25 mm; Kopf-B. 3–3,1 mm; Pronotum-L. 5,8–6 mm; B. 2–2,1 mm, Metazonen-L. 4,2–4,3 mm; Elytren-L. 15–16 mm. ♀ unbekannt.”

#### Translation.

“Galepsus (Syngalepsus) birkenmeierae n. sp. ♂. Relatively small. General colour yellowish brown, dark brown speckled. Head relatively wide, wider than pronotum. Vertex almost straight. Eyes relatively bulging laterally, dorsally rounded, without notching vertex. Lower frons wider than high. Base of antennae (flagellum) brilliant, gradually becoming darker towards the apex. Pronotum almost 3 times longer than broad, dark brown spotted or with a dark median line, metazone slightly narrower than prozone. Prosternum with a large oval black spot, which occasionally reveals the paired black spots characteristic of the subgenus. Forewings not completely covering the abdomen, just as Alae, slightly smoked. Costal field bright. Discoidal field with brown veins, quite rough, crossing anterior cubitus veins. Media and radius veins very clear and therefore interrupted. Forelegs light brown on ventral surface, with a little black spot, laterally marbled brown, trochanter with three black spots. Fore coxae almost reaching the posterior border of the prosternum. Meso- and metathoracic legs speckled brown. Cerci flat. Genitalia: ventral phallomere with short and more medial end claw, smaller subapical tooth (pda) and widely rounded corner pointing to the right. Ventral phallomere of rectangular general shape. Right phallomere, blunt, slightly thickened and with strong bristles at the apex; ventral plate (pia) with a series of granules, ventral process (pva) with a blunt basal tooth and an apical lobe. Apical process (paa) of left phallomere, slender, with the terminal part in the shape of an obtuse fork; posterior process of phalloid apophysis (apa) smooth, pointed, with a large hairy area.

Body length 24 – 25 mm; width of head 3 – 3.1 mm; pronotum length 5.8 – 6 mm, width of pronotum 2 – 2.1 mm; metazone length 4.2 – 4.3 mm; forewings length 15 – 16 mm.

♀ unknown.”

### Galepsus (Syngalepsus) beieri

Taxon classificationAnimaliaMantodeaTarachodidae

Kaltenbach, 1996

Figures 2A , 6I , 7I , 8

Galepsus (Syngalepsus) beieri : Kaltenbach 1996: 233; Ehrmann 2002: 154; Otte and Spearman, 2005: 336.

#### Holotype.

Male deposited in ZMAN Amsterdam. **Type locality**: Lake St. Lucia, False Bay, Natal, South Africa. **Paratype**: Male deposited in NHM Wien.

#### Material examined.

1 male. South Africa, Mpumalanga, Blyde River Canyon, Swadini Resort, 24°30'54.7"S, 30°48'8.64"E, 600 m, 18.XI.2017 (SA17-05 field code), Decaëns T. & Rougerie R. leg., genitalia prep. Moulin NM200 (RCNM).

#### Diagnosis.

Galepsus (S.) beieri is very similar to Galepsus (S.) bipunctatus and G. (S.) birkenmeirae. G. (S.) beieri is distinguished from G. (S.) birkenmeirae and G. (S.) denigratus by the presence of two black spots on prosternum. Kaltenbach, in 1996, speaks of not blackened pronotum in ‘Differentialdiagnose’ but he confuses with the prosternum. Right phallomere with a process on the main posterior lobe; ventral plate (pia) with a tooth turned to the right at the apex; Left phallomere with posterior process of phalloid apophysis (apa) (pseudophallus) ended with rounded apex, distal process (paa) (titillator) with apex in mallet form at the apex, covered by thick bristles.

#### Original description of Galepsus (Syngalepsus) beieri by Kaltenbach (1996: 233).

“Galepsus (Syngalepsus) beieri sp. n. (♂; ♀ unbekannt): Kopf deutlich breiter als das Pronotum. Vertex fast gerade, nur gegen die Augen zu schwach nach frontal abfallend. Komplexaugen lateral flach gekrümmt. Frontalschild 1,4mal so breit wie hoch. Antennen mit bräunlichen Basalgliedern und ockerfarbener Geißel. Pronotum 2,6–2,7mal so lang wie über den Coxen breit. Metazone etwas schmäler als Prozone. Prosternum mit paarigen schwarzen Makeln. Elytren etwa 3mal so lang wie das Pronotum, das Abdomenende nicht erreichend. Aderung hell bräunlich. Alae hyalin. Coxae und Femora der Vorderbeine ohne auffällige Flecken. Vordertibien mit 11 Außendornen. Cerci etwas abgeflacht. Gesamtfärbung bräunlich. Kopulationsorgan: Rechter Epiphallus distal verbreitert, Apex mit aufgesetztem Zapfen. Mediale Apophysenlippe mit einem großem Apikalzahn, Innenrand glatt, nur distal mit einer kurzen Reihe auf die Lippenfläche verlagerter, kleiner, breiter Zähnchen. Linker Epiphallus mit fußartigem Anhang, ähnlich wie bei manchen Arten von *Lygdamia*. Dieser Anhang ist dicht mit nadelartigen Borsten besetzt. Pseudophallus fingerartig, mit stumpfem Apex. Der Hypophallus trägt eine spitze Endklaue wie *G.bipunctatus* (Beier, 1954: fig. 4A) und *G.birkenmeierae* (Beier, 1969b: Abb. 2). Maße in mm (♂): Long. corp.: 30,0 – 31,5; Long. pronoti: 7,4 – 7,5, Lat. pronoti: 2,7 – 2,8; Long. elytr.: 21,5 – 22,0.”

#### Translation.

“Galepsus (Syngalepsus) beieri sp. n. (♂; ♀ unknown): Head significantly wider than the pronotum. Vertex almost straight, weakly collapsed near the eyes. Eyes slightly rounded laterally. Lower frons 1.4 times wider than high. Antennae with brownish flagellum (first segments) and another segments ocher. Pronotum 2.6–2.7 times longer than its width above the coxae. Metazone barely narrower than prozone. Prosternum with two paired black spots. Forewings about 3 times longer than pronotum, not reaching the end of abdomen. Brownish veins, shiny. Hindwings not coloured. Coxae and femora of forelegs without visible spots. Foretibiae with 11 posteroventral tibial spines. Cerci slightly flattened. General brownish colour. Male Genitalia: Right phallomere widened distally, apex with a process. Ventral process (pva) with a big tooth, ventral plate (pia) smooth, with just a row of small teeth, outer wall with wider teeth. Left phallomere with a mallet form apical process (paa) (titillator), similar to that in some *Lygdamia*. Apical process (paa) densely covered with hair, like needles. Posterior process of phalloid apophysis (apa) (pseudophallus) finger-shaped, with blunt end. Ventral phallomere with a pointed claw (pda) at the apex as in *G.bipunctatus* (Beier, 1954: fig. 4A) et *G.birkenmeierae* (Beier, 1969b: fig. 2). Dimensions in mm (♂): body length 30.0 – 31.5; pronotum length 7.4 – 7.5, width of pronotum 2.7 – 2.8; forewings length 21.5 – 22.0.”

The two new species have the following diagnostic characteristics of the subgenus: Always small and delicate appearance; head rounded pentagonal; vertex almost straight or only slightly convex; lower frons wider than high; Prosternum with two rounded black spots near the middle of the metazone, sometimes also largely blackened, so that the spots are occulted; wings of males not protruding abdomen, clear or slightly brownish; wings of females shortened; small dark spots on trochanter and at the base of the anteroventral femoral spines, occasionally fore coxae basally browned; left phallomere with elongated apical process (paa), more or less wide at the apex but always covered with thick silks, posterior process of phalloid apophysis (apa) short; right phallomere obtuse.

### Galepsus (Syngalepsus) bucheti sp. n.

Taxon classificationAnimaliaMantodeaTarachodidae

http://zoobank.org/369C429F-5AC5-411F-99E6-4AE232422FE9

**Figure 3.**
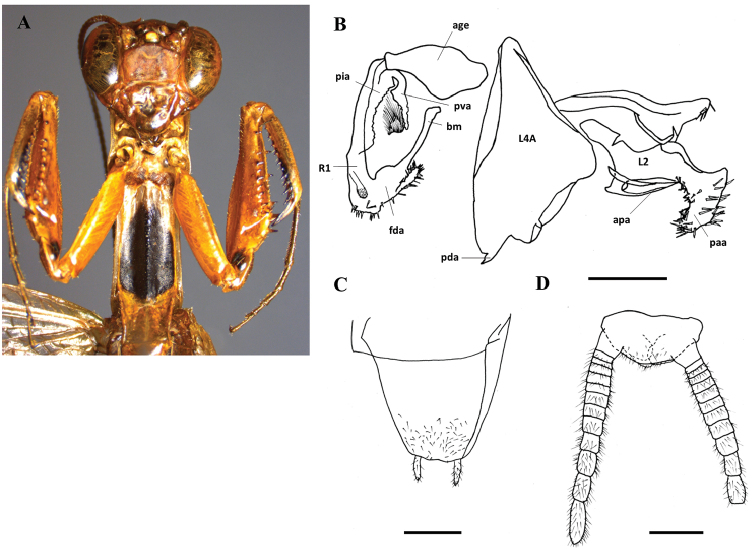
Galepsus (S.) bucheti sp. n., holotype male. **A** Prosternum and forelegs details **B** Genitalia **C** Subgenital plate **D** Cerci. For abbreviations see Brannoch et al. (2017) and the text. Scale bar: 1.00 mm.

**Figure 4.**
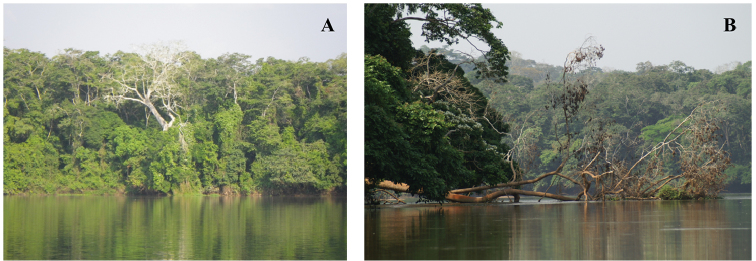
**A, B** Habitat of Galepsus (Syngalepsus) bucheti sp. n. Arboreal stratum on bank of the Sangha river in CAR. Micro-habitat couldn’t be described because the only specimen known was captured by a light trap on the river bank.

Figures 3A–D , 4A–B , 6H , 7H , 8

#### Type material.

**Holotype** male: CAR, Dzanga-Ndoki National Park, Mboki, bank of the Sangha River, 02°28'09"N, 16°04'44"E, 367m, Mercury Vapor light trap, 24.I.2012 (RP field code), N. Moulin leg., genitalia prep. Moulin NM0103 (MNHN, ex Moulin coll.), DNA barcoding BOLD NMMAN11-0518.

Unique male known.

#### Type locality.

The type specimen was collected at Mboki, on the banks of the Sangha River, the border between the Central African Republic and Cameroon, in the territory of the Dzanga-Ndoki National Park. The vegetation close to Sangha River is a mosaic of a semi-evergreen forest that contains swamp-forest areas along the rivers and shrubby areas in disturbed environments.

#### Etymology.

Named for Sergej Buchet, researcher participating at “SANGHA, Biodiversité en Terre Pygmée” on CAR, in 2008 and 2010; large contributor in the Author’s crowdfunding “A la recherche de la Biodiversité des Mantes d’Afrique”, KissKissBankBank platform (April 2015).

#### Diagnosis.

Close to Galepsus (Syngalepsus) denigratus Beier and even more to Galepsus (Syngalepsus) birkenmeierae Beier with the large black patch on prosternum but different by genitalia conformation. Head wider than the pronotum; vertex slightly convex; prosternum with a large black patch.

#### Description male.

Fine *body*, brown, length 32.7 mm from head to subgenital plate. Hind wings with venation brown. *Head*. Vertex straight or slightly convex with the region between the parietal sutures and the eyes convex. Two black spots near of the eyes, on the posterior face of the head. Lower frons transverse. Maxillary and labial palps with base of penultimate and last segment with black patch. Below of the last segment black. Antennae brown-black. *Thorax*. Pronotum. Smooth lateral margins. Surface smooth. Prozone broader than metazone. Pronotum 2.8 times longer than broad. Covered by small dark-brown spots, diffuses. Prosternum with a large black patch covering the posterior ¾ of the surface. Place of the spot within the other species visible. *Forelegs*. Femora with 12 anteroventral spines, four posteroventral spines, four discoidal spines; tibiae with eleven anteroventral spines. ten-eleven posteroventral spines. Legs globally of same colour of pronotum. Coxae without coloured patch. Trochanter with a black spot. Base of femora with a black patch, in extension of the one of trochanter. Tibiae with an elongated black patch at base of anteroventral tibial spines, from the 3^rd^ to 11^th^. All tarsomere black below. Meso- and metathoracic legs stained with dark-brown. All tarsomere black at the distal end. *Wings*. Forewings and hindwings are uniformly translucent pale brown with brown veins. Reaching tip of abdomen. *Abdomen*. Flattened. Supra-anal plate: two times wider than length, distal margin rounded, pubescent. Cerci: relatively longs, flattened, the last three distal cercomeri longer than wide. Subgenital plate: pubescent, posterior edge almost straight. Styles: long, relatively thin, hairy.

*Genitalia*. Right phallomere with ventral process (pva) and ventral plate (pia) sclerotised; posterior process of phalloid apophysis (apa) long, with sclerotised denticles, ventral plate (pia) sclerotised with a rectangular form in anterior part and a hairs tuft at the posterior part. Apex of right phallomere rectangular covered on apex and left side with thick bristles. Ventral phallomere elongated, with a tooth (pda) on posterior margin, distant of the left margin. Left phallomere with a posterior process of phalloid apophysis (apa) pointed shorter than apical process (paa) and sclerotised at apex. Apical process (paa) large, hammer form at apex. Thick bristles on posterior process of phalloid apophysis (apa) apex and main lobe (fda) of the right phallomere apex.

Female unknown.

#### Measurements (mm).

Holotype: body length 32.7, pronotum length 7.1, fore wings length 23.2, fore coxae length 4.3, fore femora length 4.9, fore tibiae length 2.9; width of pronotum 2.5, width of head 4.3, width of fore femora 1.5.

### Galepsus (Syngalepsus) dudleyi sp. n.

Taxon classificationAnimaliaMantodeaTarachodidae

http://zoobank.org/EFCF48A0-33E1-4F62-9973-CE6D7C436DDA

**Figure 5.**
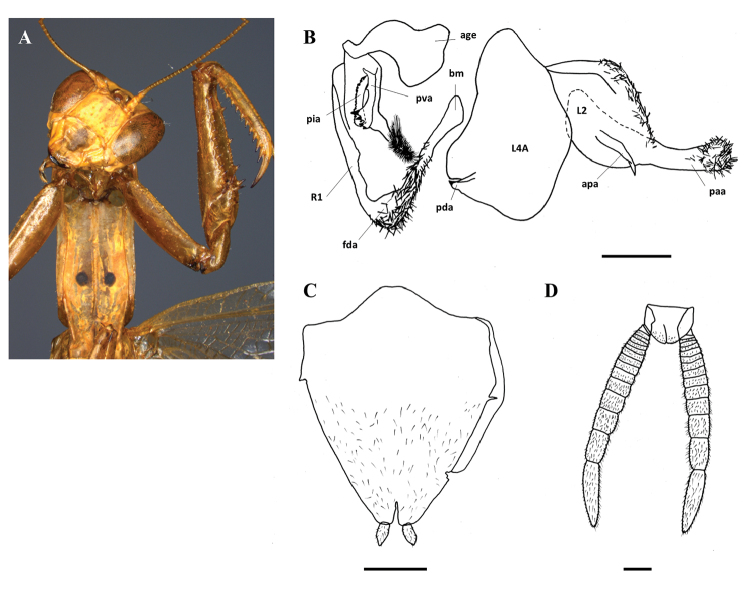
Galepsus (S.) dudleyi sp. n., holotype male. **A** Prosternum and forelegs details **B** Genitalia **C** Subgenital plate **D** Cerci. For abbreviations see Brannoch et al. (2017) and the text. Scale bar: 1.00 mm.

**Figure 6.**
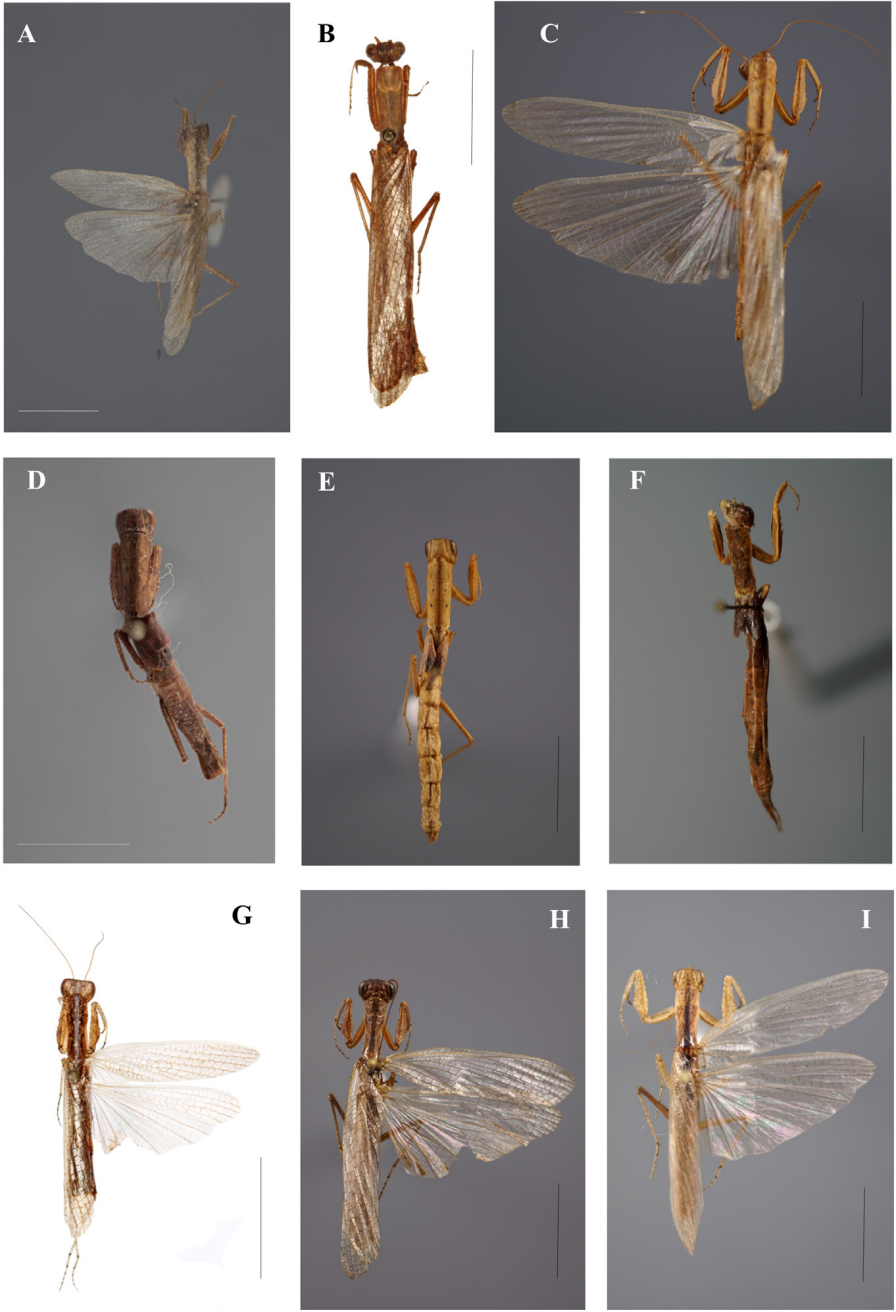
Galepsus (Syngalepsus), dorsal habitus: **A**G. (S.) bipunctatus, holotype male, Quilimane, Mozambique (G. Svenson) **B**G. (S.) denigratus, male, Kasai, Republic of Congo (H. Bruckner) **C**G. (S.) dudleyi sp. n., holotype male, Mount Soche, Malawi (N. Moulin) **D**G. (S.) denigratus, allotype female, Kasai, Republic of Congo (G. Svenson) **E, F**G. (S.) denigratus, females, Voka, Republic of Congo (N. Moulin) **G**G. (S.) birkenmeirae, holotype male, Mangochi (Fort Johnston), Malawi (H. Bruckner) **H**G. (S.) bucheti sp. n., holotype male, Mboki, Dzanga-Ndoki NP, Central African Republic (N. Moulin) **I**G. (S.) beieri, male, Mpumalanga (Swadini resort), South Africa (N. Moulin). Scale bar: 10.00 mm.

**Figure 7.**
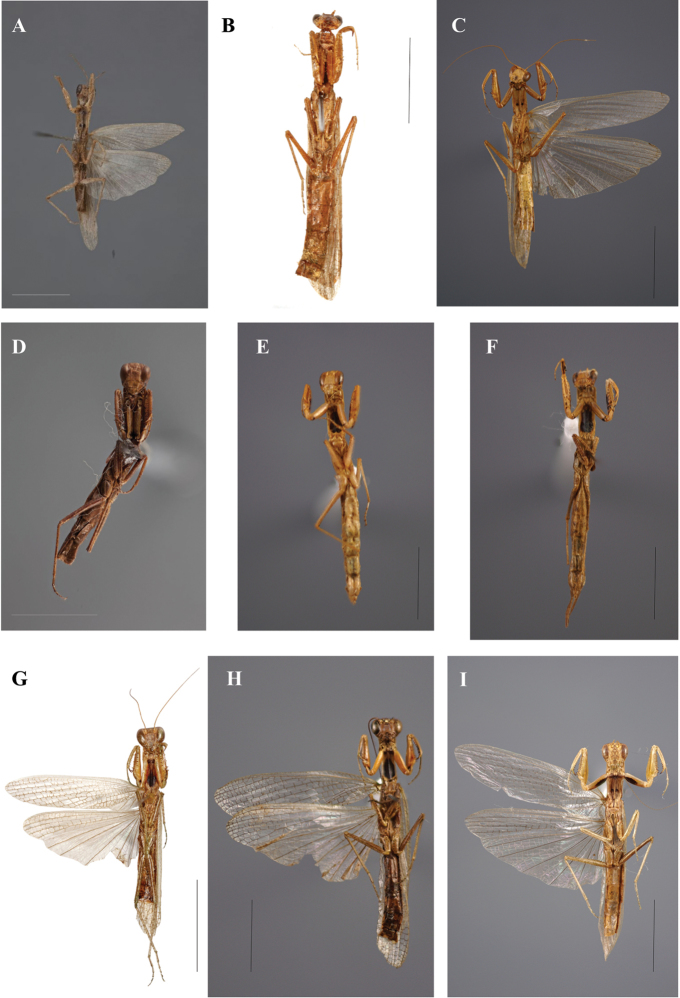
Galepsus (Syngalepsus), ventral habitus: **A**G. (S.) bipunctatus, holotype male, Quilimane, Mozambique (G. Svenson) **B**G. (S.) denigratus, male, Kasai, Republic of Congo (H. Bruckner) **C**G. (S.) dudleyi sp. n., holotype male, Mount Soche, Malawi (N. Moulin) **D**G. (S.) denigratus, allotype female, Kasai, Republic of Congo (G. Svenson) **E, F**G. (S.) denigratus, females, Voka, Republic of Congo (N. Moulin) **G**G. (S.) birkenmeirae, holotype male, Mangochi (Fort Johnston), Malawi (H. Bruckner) **H**G. (S.) bucheti sp. n., holotype male, Mboki, Dzanga-Ndoki NP, Central African Republic (N. Moulin) **I**G. (S.) beieri, male, Mpumalanga (Swadini resort), South Africa (N. Moulin). Scale bar: 10.00 mm.

**Figure 8.**
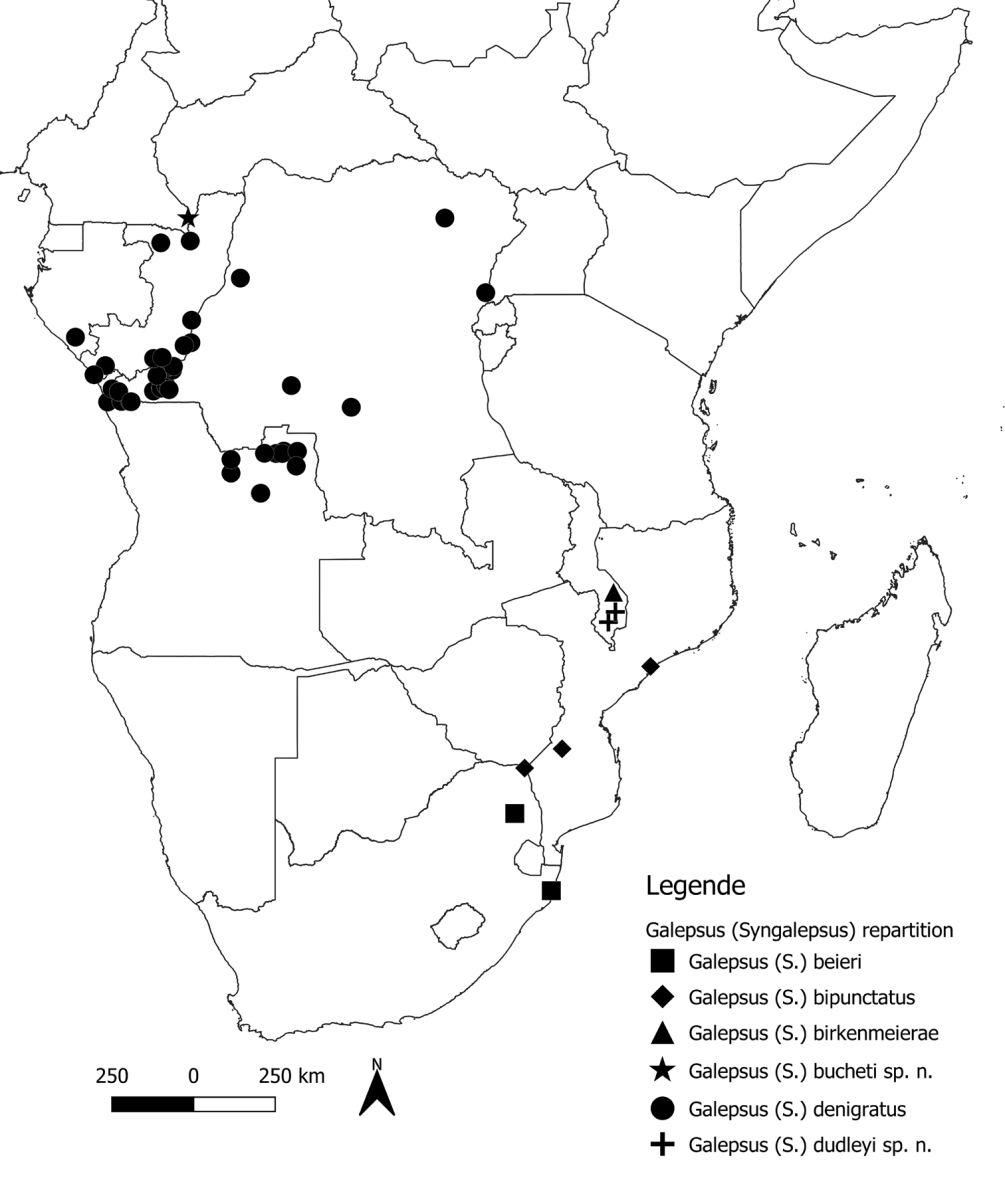
Distribution map of Galepsus (Syngalepsus) species. Source: http://www.gadm.org GADM database of Global Administrative Areas.

Figures 5A–D , 6C , 7C , 8

#### Type material.

**Holotype** male: MALAWI, Mount Soche [15°50'29.0"S - 35°01'21.0"E], Alt. 1500m, 13.I.1973, code M1787, C. O. Dudley leg., genitalia prep. Roy 3312 (MNHN). **Paratype** male: MALAWI, Zomba, Mlunguzi East [15°23'S - 35°20'E], Alt. 938m, 05.II.1975 (1♂), H. R. Feijen, genitalia prep. Roy 3575 (MNHN).

#### Type localities.

The type specimen was collected at Mount Soche, South of Blantyre, in the Southern region of Malawi. The altitude of this geological formation is approximately 1500 m. It’s surrounded by urbanization, except in the south where agricultural practices take place. Paratype was also collected in the southern region of Malawi, at Zomba, Mlunguzi East.

#### Etymology.

Named for the first collector, Cornell O. Dudley, Professor of botanic and entomology in Malawi.

#### Diagnosis.

Close to Galepsus (Syngalepsus) beieri Kaltenbach and Galepsus (Syngalepsus) bipunctatus Beier with the two black spots on prosternum but different by genitalia conformation: ventral process (pva), of right phallomere, end with several pointy teeth; left phallomere with apical process (paa) without a bump that gives a spoon or mallet appearance. Head as wide as the pronotum; vertex straight; prosternum with two black spots.

#### Description male.

Fine *body*, light brown, length 32–34 mm from head to subgenital plate. Hindwings with venation light brown. *Head*. Slightly wider than pronotum. Vertex: straight, slightly convex. Frontal shield transversal, wider than high. Labial palps with the last two segments stained black at the base on both segments, and below for the apical segment. Maxillary palps broken. Antennae light brown. *Thorax*. Pronotum, three times longer than its largest width, prozone broader than metazone, lateral margins and surface smooth; median line of the pronotum blackened. Prosternum, same colour of the pronotum, with two black spots closed to centre of prosternum, on both sides of the median line. *Forelegs*. Coxae without coloured patch, two-three very small tubercles on the anteroventral border. The dark spot on the left trochanter is not a spot of colour on the cuticle but blackened material inside the tegument. Femora: with 11–12 anteroventral spines, four discoidal spines, four posteroventral spines, all spines with black-brown apex. Tibiae: with 11–12 anteroventral spines, eleven posteroventral spines, tarsus with blackened underside. *Wings*. Forewings and hindwings are uniformly translucent pale brown with light-brown veins. Reaching tip of abdomen.

*Abdomen*. Flattened. Supraanal plate: almost wider than length, distal margin rounded, pubescent. Cerci: relatively longs, flattened, the last two cercomeri longer than wide. Subgenital plate: triangular shape, pubescent. Styles: relatively short, thick, hairy. *Genitalia*. Right phallomere with ventral process (pva) and ventral plate (pia) sclerotised; ventral process (pva) margin with large denticles. Apex of the right phallomere rounded, covered on apex and left side with thick bristles; hairs tuft at the posterior part. Ventral phallomere large, with a sharp process on posterior margin, towards the right. Left phallomere with a posterior process of phalloid apophysis (apa) long and sharp, slightly sclerotised at apex. Apical process (paa) long and round at apex, covered at apex by thick bristles; left margin of left phallomere covered by thick bristles.

Female unknown.

#### Measurements (mm).

Holotype: body length 32, pronotum length 8.6, fore wings length 24.3, fore coxae length 4.2, fore femora length 6.4, fore tibiae length 3.7; width of pronotum 2.9, width of head 3.5, width of fore femora 1.7.

Paratype: body length 33.7, pronotum length 8.0, fore wings length 26.3, fore coxae length 5.0, fore femora length 6.0, fore tibiae length 3.8; width of pronotum 3.0, width of head 4.0, width of fore femora 2.0.

##### Identification key to species of Galepsus (Syngalepsus) using males

**Table:** 

1	Prosternum with two black spots	**2**
–	Prosternum with a large black patch	**4**
2	Cerci flattened with distal cercomere long and narrow. Right phallomere with ventral process (**pva**) with denticules	**3**
–	Cerci slightly flattened with distal cercomere not long and narrow. Right phallomere with a small process at the apex and ventral process (pva) without denticules. Left phallomere with apical process (paa) shaped like a small mallet, covered of bristles	**G. (S.) beieri**
3	Right phallomere with some big teeth towards right on ventral process (pva), left phallomere with apical process (paa) not shaped like a spoon, long, with rounded apex and covered of thick bristles	**G. (S.) dudleyi sp. n.**
–	Right phallomere without big sharp teeth on ventral process (pva), but a regular rough margin. Left phallomere with apical process (paa) shaped like a spoon, with a little lateral process	**G. (S.) bipunctatus**
4	Body length more than 27 mm. Left phallomere with apical process (paa) large, covered of numerous and thick bristles	**5**
–	Body length less than 26 mm. Ventral face of forelegs with black spots on femora and trochanter. Left phallomere with apical process (paa) long and narrow, bristles only at apex	**G. (S.) birkenmeierae**
5	Forelegs without black patches on ventral face. Left phallomere with a large apical process (paa), with rounded apex, covered of thick bristles	**G. (S.) denigratus**
–	Forelegs with black patches on ventral face of femora and trochanters. Left phallomere with apical process (paa) shaped like a hammer, covered of thick bristles	**G. (S.) bucheti sp. n.**

##### Clé d’identification des espèces de Galepsus (Syngalepsus) d’après les mâles

**Table:** 

1	Prosternum avec deux points noirs	**2**
–	Prosternum avec une grande tache noire	**4**
2	Cerci aplatis avec le dernier segment long et étroit. Epiphallus droit avec le processus ventral (pva) portant des dents	**3**
–	Cerci légèrement aplatis avec le dernier segment pas long et étroit. Epiphallus droit avec un petit prolongement à son extrémité et le processus ventral (pva) sans dents. Epiphallus gauche avec l’extrémité du titillateur en forme de petit maillet couvert de soies	**G. (S.) beieri**
3	Epiphallus droit avec quelques grandes dents orientées vers la droite sur l’apophyse, epiphallus gauche avec le titillateur pas en forme de cuillère, long, avec le bout arrondi et couvert de soies épaisses	**G. (S.) dudleyi n. sp.**
–	Epiphallus droit sans de grosses dents pointues sur l’apophyse, mais une cannelure presque régulière, epiphallus gauche avec le titillateur en forme de cuillère, avec un petit prolongement latéral	**G. (S.) bipunctatus**
4	Longueur du corps supérieure à 27 mm. Epiphallus gauche avec un titillateur large, couvert de nombreuses et épaisses soies	**5**
–	Longueur du corps inférieure à 26 mm, intérieur des pattes antérieures avec des taches noires sur les fémurs et trochanters. Epiphallus gauche avec le titillateur long et fin, des soies uniquement vers l’extrémité	**G. (S.) birkenmeierae**
5	Pattes antérieures sans taches noires sur la face intérieure. Epiphallus gauche avec le titillateur large, au bout arrondi, couvert de soies épaisses	**G. (S.) denigratus**
–	Pattes antérieures avec des taches noires sur la face interne des fémurs et trochanters. Epiphallus gauche avec le titillateur en forme de marteau couvert de soies épaisses	**G. (S.) bucheti n. sp.**

##### Checklist of species of G. (Syngalepsus) (Fig. 8)

Galepsus (Syngalepsus) beieri Kaltenbach, 1996: Natal, South Africa.

Galepsus (Syngalepsus) bipunctatus Beier, 1931: Mozambique, Natal, Transvaal, South Africa.

Galepsus (Syngalepsus) birkenmeierae Beier, 1969b: Malawi.

Galepsus (Syngalepsus) bucheti sp. n.: Dzanga-Ndoki National Park, Central African Republic.

Galepsus (Syngalepsus) denigratus Beier, 1954: Angola, Democratic Republic of Congo, Gabon, Republic of the Congo, Uganda.

Galepsus (Syngalepsus) dudleyi sp. n.: Malawi.

## Discussion

The present study led to the description of two new species, Galepsus (Syngalepsus) bucheti sp. n. and Galepus (Syngalepsus) dudleyi sp. n. Males are easily identifiable using the characters of both habitus and male genitalia. However, females are unknown, which is common for mantises because studies with light trap sampling fail to attract females. Here, only females of G. (S.) denigratus are known (Beier 1969a). They are brachypterous and likely live on tree trunks and branches. Sweep nets and visual searching should be used more broadly in Mantodea studies to ensure more females can be collected. Furthermore, this method will also reveal mantises’ micro-habitat preferences. In CAR, no female of G. (S.) bucheti sp. n. were collected, although all known modern sampling techniques were implemented (sweeping, beating, tree climbing, light trap). With brachypterous females it a likely the distribution of each species could be restricted to small geographic ranges, which could indicate many species remain to be discovered in remote areas of Africa and other parts of the world.

## Supplementary Material

XML Treatment for Galepsus (Syngalepsus) bipunctatus

XML Treatment for Galepsus (Syngalepsus) denigratus

XML Treatment for Galepsus (Syngalepsus) birkenmeierae

XML Treatment for Galepsus (Syngalepsus) beieri

XML Treatment for Galepsus (Syngalepsus) bucheti

XML Treatment for Galepsus (Syngalepsus) dudleyi
